# Comparison of *In Situ* Polymerization and Solution-Dispersion Techniques in the Preparation of Polyimide/Montmorillonite (MMT) Nanocomposites

**DOI:** 10.3390/ijms12096040

**Published:** 2011-09-19

**Authors:** Mansor Bin Ahmad, Yadollah Gharayebi, Mohd. Sapuan Salit, Mohd. Zobir Hussein, Kamyar Shameli

**Affiliations:** 1 Department of Chemistry, Universiti Putra Malaysia, 43400 UPM Serdang, Selangor, Malaysia; E-Mails: mzobir@fsas.upm.edu.my (M.Z.H.); kamyarshameli@gmail.com (K.S.); 2 Advanced Materials and Nanotechnology Laboratory, Institute of Advanced Technology (ITMA), Universiti Putra Malaysia, 43400 UPM Serdang, Selangor, Malaysia; E-Mail: masoud.gharayebi@gmail.com; 3 Department of Chemistry, Islamic Azad University Behbahan Branch, University Street, Behbahan, 6361713198, Iran; 4 Department of Mechanical and Manufacturing Engineering, Universiti Putra Malaysia, 43400 UPM Serdang, Selangor, Malaysia; E-Mail: sapuan@eng.upm.edu.my

**Keywords:** polyimide, Organo Montmorillonite, nanocomposites, *in situ* polymerization, solution-dispersion

## Abstract

In this paper, Polyimide/Montmorillonite Nanocomposites (PI/MMT NCs), based on aromatic diamine (4-Aminophenyl sulfone) (APS) and aromatic dianhydride (3,3′,4,4′-benzophenonetetracarboxylic dianhydride) (BTDA) were prepared using *in situ* polymerization and solution-dispersion techniques. The prepared PI/MMT NCs films were characterized by X-ray diffraction (XRD), Fourier transform infrared spectroscopy (FT-IR), transmission electron microscopy (TEM) and thermogravimetric analysis (TGA). The XRD results showed that at the content of 1.0 wt % Organo Montmorillonite (OMMT) for two techniques and 3.0 wt % OMMT for the *in situ* polymerization technique, the OMMT was well-intercalated, exfoliated and dispersed into polyimide matrix. The OMMT agglomerated when its amount exceeded 10 wt % and 3.0 wt % for solution-dispersion and *in situ* polymerization techniques respectively. These results were confirmed by the TEM images of the prepared PI/MMT NCs. The TGA thermograms indicated that thermal stability of prepared PI/MMT NCs were increased with the increase of loading that, the effect is higher for the samples prepared by *in situ* polymerization technique.

## 1. Introduction

Among many industrial polymers, polyimides (PIs) show excellent thermal stability, chemical resistance, low dielectric constant, but high mechanical properties [1]. Therefore, they have extensively been used in various industrials, such as microelectronics, optical, automobile, and aerospace as composite matrices, adhesives, coatings, fibers, foams, membranes, and films [2]. There are many synthetic methods available for the preparation of PIs. However the most important alternative is the reaction of an aromatic dianhydride with an aromatic diamine to form soluble precursor, poly(amic acid) (PAA), which is subsequently, chemically or thermally converted into PI [3].

There are many studies which show clay can greatly improve PI properties [4–7]. In fact, the microstructure and dispersion of clay into the PI matrix increases prepared PI/clay nanocomposites (NCs) performance [8]. The Toyota Research Group has successfully prepared PI/clay NCs through the two-step polymerization of PI which is based on pyromellitic dianhydride (PMDA) and oxydianiline (ODA) [9]. The performance of polyimide/clay nanocomposite varies depending on the dispersion of clay in the polyimide matrix, the type of polyimide, the alkyl ammonium ions and the silicates present in the clay [10,11].

A number of attempts have been carried out to disperse clay into polyimide matrix and increase the performance of polyimide/clay NCs [10]. The montmorillonite (MMT) is a kind of clay which is most commonly used in the preparation of PI/MMT NCs. The dimension of the MMT is about 100–218 nm in length and 1 nm in thickness [12]. The MMT is a hydrophilic compound which can be converted to an organophilic compound in order to make miscible with organic polymers by ion-exchange reaction. The sodium ions in the layer gallery were replaced with organic molecules which contained ammonium ions [13]. In many studies, organoclay was proved to not fully disperse in the polymer matrix [14–16]. In general, three methods have commonly been used to organically disperse modified layered silicates into a polymer. The first approach is the *in situ* polymerization method [17], where polymerization occurs in the gallery located between the silicate layers. However, this approach is not generally applicable to all the polymer types and polymerization is often incomplete. The second method involves dispersion of a layered silicate suspension in a polymer solution by high shaking [18]. Although this particular method is rather simple, the exfoliated layered silicates are often stacked during solvent evaporation or thermal cure. The third approach involves melt blending under high shear [14,19] and it is primarily applicable to thermoplastic polymers. Nonetheless, these methods can not completely disperse the clay in a polymer matrix due to the intrinsic strong attraction among the clay layers with large surface areas.

In the present study, a novel PI/MMT NCs were synthesized and characterized using the *in situ* polymerization and solution-dispersion techniques. In addition, the dispersion content of clay in PI matrix and the thermal stability of the prepared PI/MMT NCs using both techniques were also compared.

## 2. Results and Discussion

### 2.1. X-ray Diffraction and Thermogravimetric Analysis (TGA) Study of OMMT Structure

**Figure 1:**
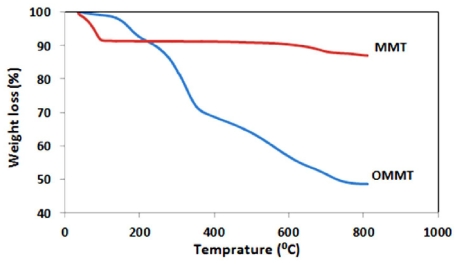
Thermogravimetric analysis (TGA) Thermograms of the Montmorillonite (MMT) and Organo Montmorillonite (OMMT).

**Figure 2:**
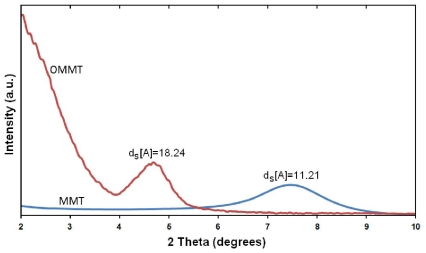
X-ray diffraction (XRD) patterns of MMT and OMMT.

The MMT is a hydrophilic component. Thus modification of the MMT for dispersion in organic solvents and organic polymer materials is necessary. Many researchers have reported that the distances of the interlayer galleries in stacked silicate sheets of the MMT clay is increased by using of a positively charged organic surfactant. The positive ions (Na^+^) in the structure of the Na-MMT clay can be replaced with positive organic surfactant ions by ion exchange process. This replacement enhances the interaction of the modified silicate sheets with organic solvents and organic polymers. Consequently, the packed silicate sheets of the MMT are allow to enter the polymer chains in the interlayer galleries (*i.e.*, intercalation) and can also expand without any interaction with other species of the MMT (*i.e.*, exfoliation). The needed driving forces for polymer chains to enter the interlayer galleries are generally unfavorable because there is more than one MMT particle that can be associated with the same surfactant molecule. This can prevent making a suitable exfoliation of MMT. Thus, for prevention of this problem a surfactant is chosen with a long non-polar chain. On the other hand, this kind of surfactant can increase the distances of MMT sheets [20]. In this study, modification of the MMT was carried out with octadecyl amine (ODA), *i.e.*, a long non-polar chain aliphatic surfactant. Meanwhile, the Na-MMT and organically modified MMT (OMMT) product were characterized using thermogravimetric analysis (TGA) and X-ray diffraction (XRD). The TGA thermograms obtained on the MMT and modified MMT (OMMT) in nitrogen atmosphere are shown in Figure 1. The OMMT began to lose mass around 165 °C, and lost approximately 40 wt % after running until 600 °C. This is probably due to the loss of ODA grafted on silicate sheets of MMT and more specifically the degradation of the hydrocarbon chains. The structure of Na-MMT and organically modified MMT (OMMT) were characterized using X-ray diffraction (XRD). The XRD patterns of MMT and OMMT are shown in Figure 2. The XRD measurements showed that 2θ had been shifted from the high angle to the low angle after the modification of MMT to afford OMMT by ion exchange process. This shift indicated the interlayer spacing of MMT (2θ = 7.12; *d* = 11.2 Å) was increased to afford OMMT (2θ = 4.83; *d* = 18.2 Å). This observation could be explained by Bragg’s equation, indicating that octadecyl ammonium (ODA) was intercalated into the MMT galleries, and ODA might adopt a bilayer structure parallel to adjacent silicate layers in the interlayer space [21].

### 2.2. Chemical Analysis of PAA and PI by Fourier Transform Infrared Spectroscopy

**Figure 3:**
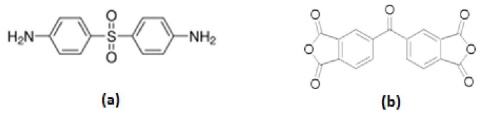
The Structure of: (**a**) 4-aminophenyl sulfone (APS); and (**b**) 3,3′,4,4′-benzophenonetetracarboxylic dianhydride (BTDA).

**Figure 4:**
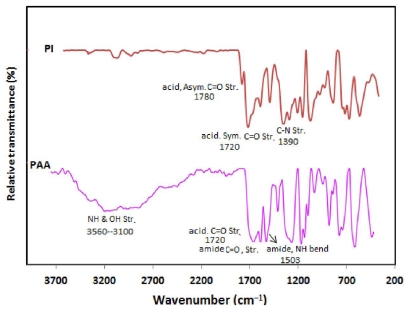
Representative Fourier transform infrared (FTIR) spectra of poly(amic acid) (PAA) and Polyimide (PI).

The polyimide (BTDA-APS), was synthesized using monomers of 3,3′,4,4′-Benzophenontetracarboxylic dianhydride (BTDA) as aromatic dianhydride and 4-Aminophenyl sulfone (APS) as aromatic diamine and their structures are shown in Figure 3. For the synthesis of polyimide (BTDA-APS), aromatic dianhydride BTDA was reacted with aromatic diamine APS, which produced soluble PAA as a precursor (stage 1) and, in which the prepared PAA was subsequently thermally converted into PI (BTDA-APS) (stage 2). At the same time, the Fourier transform infrared (FTIR) spectroscopy confirmed the formation of PAA. Typical FTIR spectra of PAA and PI, which was prepared from thermal imidization of PAA, are shown in Figure 4. Figure 4 illustrates the characteristics of amide absorption bands at 3200–3470 cm^−1^, 1720 cm^−1^, 1660 cm^−1^ and 1503 cm^−1^ corresponding N–H and O–H stretching, acid, C=O stretching, amide, C=O stretching and N–H bending, respectively. In addition, the FTIR spectroscopy also confirmed the conversion of PAA into PI. After the thermal imidization of PAA, the characteristic of amide absorption bands for PAA was found to have disappeared, while several new absorption bands corresponding to PI [22] appeared near 1780 cm^−1^, 1720 cm^−1^, 1390 cm^−1^ and 745 cm^−1^ corresponding asymmetric C=O stretching, symmetric C=O stretching, C–N stretching and imide ring deformation, respectively as was been shown at the FTIR spectra of PI.

### 2.3. Chemical Analysis of PI/MMT NC by Fourier Transform Infrared Spectroscopy

**Figure 5:**
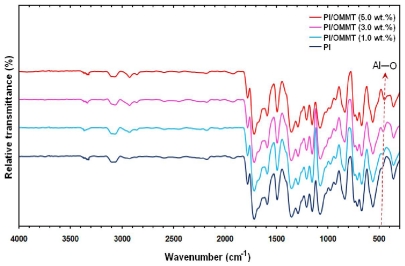
FTIR spectrums of Polyimide/Montmorillonite Nanocomposites (PI/MMT NCs) with different OMMT contents.

The PI/MMT NCs films with various contents of OMMT (0–10 wt %) were prepared to obtain the products from both the techniques. The FTIR spectra of the PI/MMT NCs with various OMMT contents were found to be similar for the two techniques shown in Figure 5. Meanwhile, the typical vibration bands of MMT were revealed at 1044 cm^−1^ (Si–O), 515 cm^−1^ (Al–O) and 465 cm^−1^ (Mg–O). The intensity of the vibration bonds with the increasing of the OMMT content in the PI nanocomposites became stronger in the FTIR spectra of PI/MMT NCs.

### 2.4. X-ray Diffraction Study of PI/MMT NCs Structure

**Figure 6:**
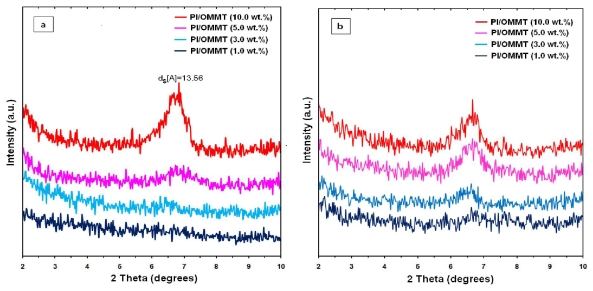
XRD diagrams of the PI/MMT NCs with different OMMT contents: (**a**) by *in situ* polymerization technique; and (**b**) by solution-dispersion technique.

The XRD patterns of the prepared PI/MMT NCs films with various OMMT contents using the *in situ* polymerization technique are shown in Figure 6(a). Nonetheless, no peak was found below 2θ = 10 for the prepared nanocomposites with 1 wt % and 3% OMMT. These results indicated that the silicate layers for the MMT were intercalated, exfoliated and dispersed in the polyimide matrix. The XRD patterns of the prepared PI/MMT NCs showed that the intensities of the PI/MMT NCs films increased with the increase of the OMMT content. Meanwhile, when the OMMT content increased more than 5 wt %, the diffraction peak of MMT at 2θ = 7.0 was visible. The XRD patterns of the prepared PI/MMT NCs films, with various OMMT contents using solution-dispersion technique, are shown in Figure 6(b). However, there was no peak below (2θ = 10) for the PI/MMT NCs film of 1 wt % OMMT content. On the contrary, when the OMMT content increased (≥3% OMMT), a peak at 2θ = 6.68 was visible. A comparison of the XRD patterns of the prepared PI/MMT NCs films using the two techniques indicated that the OMMT contents could be further dispersed into polyimide matrix using the *in situ* polymerization technique.

### 2.5. Transmission Electron Microscopy (TEM) Images of PI/ MMT NCs Film

**Figure 7:**
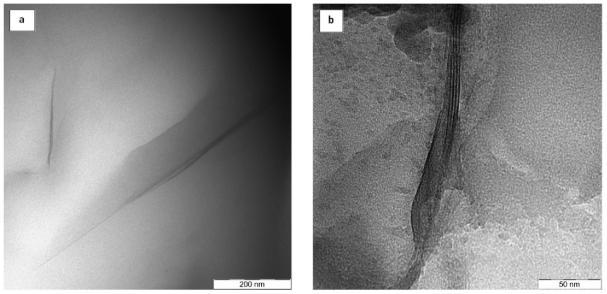
TEM image of prepared PI/(3%)MMT NC: (**a**) by *in situ* polymerization technique; and (**b**) by solution-dispersion technique.

The TEM images of the prepared PI/MMT NCs for the two techniques are shown in Figure 7. These images confirmed the exfoliated dispersion of the OMMT contents in the PI matrices by *in situ* polymerization technique is higher than the solution-dispersion technique.

### 2.6. Thermal Properties Study of PI/MMT NCs

**Figure 8:**
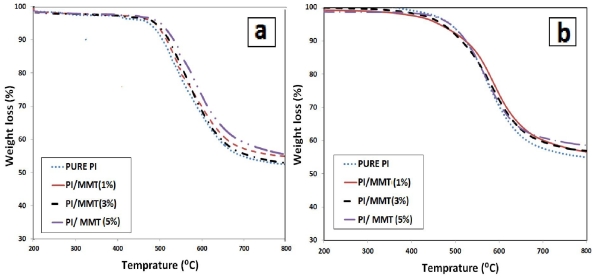
TGA Thermogram of the PI/MMT NCs with different OMMT contents: (**a**) by *in situ* polymerization technique; and (**b**) by solution-dispersion technique.

**Figure 9:**
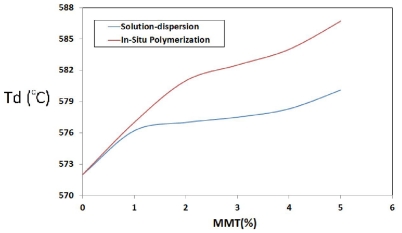
Comparison of thermal stability of the prepared PI NCs for two techniques.

Thermal properties of prepared PI/MMT NCs with two techniques were investigated by thermogravimetric analysis (TGA). Figure 8(a) and 8(b) show typical TGA thermograms of the prepared PI/MMT NCs with various OMMT contents using the *in situ* polymerization technique and the solution-dispersion technique as measured under nitrogen atmosphere respectively. It was shown that the thermal stability increased with the OMMT loading for the two techniques. The improved thermal stability in polymer/clay NCs such as prepared PI/MMT NCs is principally due to the formation of char which prevents the out-diffusion of the volatile decomposition products. The initial degradation of aromatic PI is accompanied by the volatilization of CO_2_. The presence of MMT nano-layers dispersed homogeneously, in structure of prepared PI/MMT nanocomposite films, can prevent the permeability of volatile decomposition product from the PI [23]. With increase of MMT loading, the amount of exfoliated MMT in polymer matrices is increased; therefore, the thermal stability of prepared PI/MMT NCs with two techniques is also increased. The decomposition temperature (*T*_d_) for both techniques are compared in Figure 9. As illustrated in Figure 9, the decomposition temperature (*T*_d_) for the prepared PI/MMT NCs using the *in situ* polymerization was found to be more than the prepared PI/MMT NCs by solution-dispersion. This finding indicates that there is more homogeneous dispersion of the MMT in PI matrices using *in situ* polymerization technique than with the solution-dispersion technique.

## 3. Experimental

### 3.1. Materials

4-Aminophenyl sulfone (APS) [97%] as diamine and 3,3′,4,4′-Benzophenontetracarboxylic dianhydride (BTDA) [96%] as dianhydride were obtained in the polymer grade purity from Sigma-Aldrich, St. Louis, MO, USA, and they were used as the monomers without further purification. *N*-Methyl-2-pyrrolidinone (NMP) [99.5%, extra pure, b.p. 202 °C], which was obtained from Acros Organics, was used as the solvent. Meanwhile, montmorillonite (MMT), with the cation exchange capacity of 119 meq/100 g, was obtained from Kunimine Industries Co., Tokyo, Japan. Octadecylamine (ODA) [Acros Organics, Geel, Belgium, 98% purity] was used as the modification reagent of MMT.

### 3.2. Modification of MMT

The organophilic MMT was prepared by a cationic exchange reaction between the sodium cations of MMT and quaternary alkylammonioum cations. The MMT was added to the distilled water in a round-bottom flask that was equipped with a mechanical stirrer until the MMT was dispersed in the distilled water. After that, the prepared quaternary alkylammonioum solution was then added to the MMT solution, and the mixture was vigorously stirred for 1 h. The prepared OMMT was collected by filtration and washed with water several times until no chloride ion was detected by AgNO_3_ solution (0.1 N). This was followed by drying the product in a vacuum oven for 48 h. The amount of ODA grafted onto the layered silicate of MMT surfaces was estimated 18.7% based on the total charge on the MMT layers.

### 3.3. Preparation of the PI/MMT NCs via *In Situ* Polymerization

For the preparation of the PI/MMT NCs using the *in situ* polymerization technique, in round-bottomed flask that was equipped with a mechanical stirrer, an appropriate amount of OMMT with respect to PI by 1, 3, 5 and 10 wt % were introduced into 6 g of NMP under stirring 24 h at room temperature. 1 mmol of diamine monomer APS was subsequently added into the OMMT suspension and this mixture was then stirred for 12 h more. After that, 1 mmol of dianhydride monomer BTDA was gradually added into the APS and OMMT suspension. The two different monomers solution was stirred further in room temperature (RT) for 24 h so as to produce a PAA solution. The prepared PAA/OMMT suspension was subsequently cast onto a clean glass plate. The cast films were placed in an oven under air atmosphere and heated at 80 °C for 5 h, 125 °C for 2 h, 150 °C for 2 h, 180 °C for 1 h, 200 °C for 1 h, 250 °C for 1 h and 300 °C for 0.5 h. The prepared PI/MMT NCs films appeared to be transparent and yellowish or brownish, just like pure PI film.

### 3.4. Preparation of PI/MMT NCs via Solution-Dispersion Technique

For the preparation of polyimide/MMT NCs using the solution-dispersion technique in round-bottomed flask fitted with a mechanical stirrer, the OMMT content was first calculated by 1, 3, 5 and 10 wt %, with respect to the PI which was introduced into the NMP under a 24 h magnetic stirring at room temperature. This suspension was then added into the prepared PAA solution in the NMP to form 15 wt % of the PAA and the mixture was further stirred for 24 h more. After that, the prepared PAA/OMMT suspensions were cast onto the glass plate; the thermal imidization reaction was then performed under the condition as explained in sub-section 3.3.

### 3.5. Characterization

The products of the prepared PAA and PI were characterized by FT-IR spectra (Perkin–Elmer Model: 100 Series). The MMT layers separation and interlamellar exfoliation were investigated using X-ray diffractometer (Shimadzu, Model XRD 6000). In addition, the interlamellar space was calculated from diffraction angle (e.g., 2θ) at a fixed wave length (*k*) According to Bragg equation, 2*d*sinθ = *n*λ. A wave-length (λ) of 1.5418 Å was used for these measurements. The XRD patterns were recorded at a scan speed of 4/min. The thermal properties were determined using the thermogravimetric analysis (Perkin–Elmer, Model TGA–7). The transmission electron microscopy (TEM) images were obtained using the H-7100 electron microscope (Hitachi, Tokyo, Japan).

## 4. Conclusions

The PI/MMT NCs which were prepared based on BTDA/APS polyimide using the two techniques have the following characteristics:

1. The dispersion of MMT in the PI matrices by *in situ* polymerization technique is higher than solution-dispersion technique.
2. The PI/MMT NCs have higher thermal stability compared to pristine PI.
3. The thermal stability increased with the OMMT loading for both techniques; however, this factor is higher for the *in situ* polymerization techniques.
